# Charles Henry Fox

**Published:** 1915-12

**Authors:** 


### CHARLES HENRY FOX, M.D.

The death of Dr. Charles Henry Fox, which occurred on Christmas Day, 1915, leaves his cousin, Dr. Arthur Fox, of Bath, the only medical representative of a family who have been closely associated with medical work in and around Bristol since the first Dr. Edward Long Fox became Physician to the Bristol Royal Infirmary in 1786. He was born at Brislington House on June 7th, 1837, and was the fourth son of Dr. Francis Ker Fox, of Brislington Asylum, and brother of the late Dr. Edward Long Fox, in whose memory the Long Fox Lecture was founded.

Dr. C. H. Fox was educated at Shrewsbury and Highgate College, and entered as a student at St. George's Hospital in 1855. Having obtained the M.R.C.S., he spent a year at the Edinburgh Medical School, a year which he always loved to look back upon and described as the happiest of his life. He graduated as M.D. of St. Andrews in 1860, and the following year joined his father at Brislington Asylum, of which he became a partner in 1873, and spent the whole of his life there as a medical practitioner, first with his father and latterly with his half-brother, the late Dr. Bonville Bradley Fox.

In 1894, after thirty-three years of practice, he retired and spent the remaining twenty-one years of his life in Edinburgh, to which he was drawn by his many friendships and early associations. In 1900 he was elected a Fellow of the Royal College of Physicians, Edinburgh ; he was also a Fellow of the Botanical Society of Edinburgh and of the Antiquarian Society of Scotland. With a keen sense of humour, he was a genial conversationalist.

For some years he was an almost complete invalid, but his courage was great, and despite his afflictions he was ever cheerful, his old friends rallying round him especially during the latter years of his life when his health became impaired.

He leaves behind many relatives to mourn him, and to whom we offer our sincere condolences.

